# Mapping the Neuroprotective Landscape of Perioperative Magnesium Sulphate: A Translational Scoping Review

**DOI:** 10.3390/jcm15135032

**Published:** 2026-06-28

**Authors:** Khairunnisai Tarimah, Iwan Fu’adi, Elvan Wiyarta, Lisda Amalia, Tatang Bisri, Dewi Yulianti Bisri

**Affiliations:** 1 Doctoral Study Program in Medical Science, Faculty of Medicine, Universitas Padjadjaran, Bandung 45363, Indonesia; 2 Department of Anesthesia and Intensive Care, Faculty of Medicine, Universitas Islam Al Azhar Mataram, Mataram 83237, Indonesia; 3 Division of Neuroanesthesia and Critical Care, Department of Anesthesia and Intensive Care, Faculty of Medicine, Universitas Padjadjaran, Bandung 45363, Indonesia; 4 Department of Neurology, Faculty of Medicine, Universitas Indonesia, Kota Depok 16424, Indonesia; elvan.wiyarta@ui.ac.id; 5 Department of Neurology, Faculty of Medicine, Universitas Padjadjaran, Bandung 45363, Indonesia; lisda@unpad.ac.id; 6 Faculty of Medicine, Universitas Jendral Achmad Yani, Bandung 40525, Indonesia

**Keywords:** magnesium, neuroprotection, perioperative, analgesia, inflammation

## Abstract

**Background/Objectives**: Perioperative brain injury arises from interacting pathways including excitotoxicity, neuroinflammation, and endothelial dysfunction, with limited effective pharmacological neuroprotection. Magnesium sulphate has multimodal biological effects that may address these pathways, but its translational role remains unclear. We aimed to map the translational evidence landscape of perioperative magnesium sulphate and evaluate its translational evidence profile across mechanistic, indirect, and direct clinical domains with respect to potential neuroprotective signalling. **Methods**: A scoping review was conducted following PRISMA ScR. The literature from PubMed, Scopus, the Cochrane Library, and ProQuest was screened using a Population–Concept–Context framework. Eligible studies included randomised trials, observational studies, and evidence syntheses evaluating perioperative magnesium sulphate. Evidence was categorised into direct neurological outcomes, indirect clinical outcomes, biomarker evidence, and mechanistic domains. **Results**: Eighteen studies were included, comprising randomised trials, observational studies, and reviews. Magnesium was consistently associated with reductions in postoperative pain and opioid consumption and improvements in recovery characteristics and shivering prevention. In contrast, direct neuroprotective outcomes such as cognitive function, cerebral oxygenation, and neurovascular events showed limited and heterogeneous evidence. Mechanistic mapping suggested effects on NMDA receptor modulation, calcium regulation, sympathetic tone, and endothelial stability. **Conclusions**: Perioperative magnesium sulphate demonstrates consistent indirect benefits related to analgesia and recovery but lacks robust evidence for direct neuroprotection. Its role is best conceptualised as a multimodal modulator of perioperative neural stress rather than a definitive neuroprotective agent. Future studies should adopt multidomain outcome frameworks integrating mechanistic and clinical endpoints to better define its translational impact.

## 1. Introduction

Perioperative brain injury remains a major challenge in contemporary anaesthesia because surgery and anaesthetic exposure can trigger a coordinated biological response involving excitotoxicity, neuroinflammation, endothelial dysfunction, and disruption of cerebral homeostasis that extends beyond the intraoperative period [1,2]. Perioperative neurocognitive disorders are now framed as a spectrum that includes postoperative delirium, delayed neurocognitive recovery, and postoperative neurocognitive disorder rather than a single isolated complication [3,4]. This broader framework matters because postoperative delirium and related neurocognitive disorders are common in surgical patients, especially in older adults and in those with limited physiological reserve [5,6]. Increasing evidence indicates that neuroinflammation is central to this process, with peripheral surgical stress capable of activating microglia, altering synaptic function, and propagating inflammatory signalling across the neurovascular unit [5,7,8]. Blood–brain barrier injury appears to amplify this vulnerability by facilitating peripheral to central inflammatory crosstalk and destabilising the cerebral microenvironment during the perioperative period [2,9]. Taken together, these observations explain why pharmacological neuroprotection remains an attractive translational goal but has been difficult to establish clinically, since the biological substrate of perioperative injury is distributed across several interacting pathways rather than a single target [1].

Against this background, magnesium sulphate is an especially attractive candidate because it is inexpensive, widely available, familiar in perioperative practice, and mechanistically linked to several pathways relevant to neural protection [10,11,12]. Its biological rationale is broad. Magnesium modulates N methyl D aspartate receptor activity and calcium influx, making it relevant to excitotoxic stress, neuronal calcium overload, and central sensitisation [11,12]. It has also been associated with effects on sympathetic tone, vascular reactivity, and endothelial biology, which extend its theoretical relevance beyond analgesia alone and toward preservation of perioperative cerebrovascular stability [2,11]. This multimodal profile is important because perioperative brain vulnerability is unlikely to be mitigated by agents that act on a single pathway alone [5]. A drug that can influence nociceptive amplification, inflammatory signalling, vascular tone, and recovery physiology may therefore hold translational value even when direct neurological endpoints are not uniformly improved in every clinical study. It should be emphasised, however, that the presence of these indirect perioperative effects does not in itself constitute direct neuroprotection; rather, they represent mechanistically plausible contributors to a broader neuroprotective rationale that requires validation through dedicated neurological outcome studies [1,10]. A further reason magnesium sulphate deserves dedicated translational appraisal is that perioperative neuroprotection is unlikely to be captured fully by a single endpoint alone [1,10]. In real perioperative settings, clinically meaningful protection may instead be expressed through a constellation of linked effects, including attenuation of nociceptive load, reduction in opioid exposure, preservation of haemodynamic stability, mitigation of emergence disturbance, and modulation of vascular- or barrier-related stress responses [1,10]. This is especially relevant because perioperative neurological injury often evolves through interacting mechanisms rather than a single dominant lesion, so indirect perioperative benefits may still carry mechanistic significance when interpreted within an integrated biological framework [2,5]. Emerging evidence in neuroanaesthesia also suggests that magnesium may influence markers related to blood–brain barrier permeability and selected postoperative cognitive domains, although these signals remain preliminary and insufficient to define a consistent direct neuroprotective effect [13]. For that reason, the key question is not simply whether magnesium improves one isolated recovery variable but whether the overall pattern of mechanistic, physiological, and clinical signals supports a coherent neuroprotective rationale across perioperative care [1,11].

The clinical literature partly supports this broader view, although it remains fragmented. Meta-analyses have shown that perioperative intravenous magnesium can reduce postoperative opioid consumption and improve postoperative pain outcomes across several surgical settings [14,15,16]. This analgesic relevance is particularly pertinent in the neurosurgical context, where postcraniotomy pain is reported by the majority of patients and pharmacological pain prevention remains an active area of clinical research [17]. Umbrella-level evidence has likewise identified recurrent analgesic benefit while also underscoring substantial heterogeneity in populations, protocols, and certainty of effect [10,18]. More recent synthesis suggests that intravenous magnesium may also improve the quality of postoperative recovery, raising the possibility that its effects extend beyond pain control alone [19]. Contemporary randomised trials that are also included in the present review have reported reduced emergence agitation and better early recovery-related outcomes in patients undergoing thoracoscopic lobectomy and radical mastectomy [20,21]. However, the field still lacks a coherent synthesis that interprets these signals within a translational neuroprotection framework rather than treating them solely as isolated analgesic or recovery outcomes [1,10]. That gap is important because recurrent indirect signals linked to magnesium, including reduced nociceptive burden, lower opioid exposure, smoother recovery, and possible vascular modulation, may represent components of a broader neuroprotective profile even though direct neurological evidence remains limited and heterogeneous [1,2,10]. For that reason, a scoping review is particularly well suited to the current literature because it allows for structured mapping of randomised trials, observational studies, evidence syntheses, and conceptual reviews across direct neurological outcomes, indirect perioperative outcomes, and mechanistic domains [22]. Accordingly, the present review aims to map the translational evidence landscape of perioperative magnesium sulphate and to clarify how mechanistic plausibility, indirect clinical benefit, and direct neurological outcomes collectively inform a preliminary translational framework for perioperative magnesium sulphate while explicitly acknowledging the boundary between indirect perioperative modulation and validated neuroprotective efficacy.

## 2. Methods

### 2.1. Study Design

This study was conducted as a scoping review to map the breadth and characteristics of evidence regarding perioperative magnesium sulphate administration during perioperative anaesthetic care and its potential clinical and mechanistic implications. The review was designed and reported in accordance with the Preferred Reporting Items for Systematic Reviews and Meta-Analyses extension for Scoping Reviews (PRISMA ScR) guideline [22]. The completed PRISMA-ScR checklist is provided as Supplementary Table S1.

A scoping methodology was chosen because the literature spans heterogeneous study designs, perioperative contexts, and outcome domains, including clinical, biomarker-related, and mechanistic evidence. Accordingly, the objective of this review was to characterise the evidence landscape and identify dominant outcome domains rather than to estimate pooled treatment effects. This review was not registered in a prospective review registry, and therefore, no registration number is available.

### 2.2. Eligibility Framework

Eligibility criteria were structured using the Population–Concept–Context framework recommended for scoping reviews [23]. The population comprised human patients undergoing surgical procedures requiring perioperative anaesthetic management. The concept of interest was perioperative administration of magnesium sulphate as an anaesthetic adjuvant and its association with clinically relevant outcomes or mechanistically relevant pathways. Outcomes of interest included postoperative pain, anaesthetic or opioid consumption, recovery characteristics, haemodynamic responses, shivering, neurological outcomes, cerebral oxygenation, inflammatory signalling, endothelial function, and related pharmacodynamic mechanisms such as N methyl D aspartate receptor modulation or calcium channel regulation. The context was perioperative anaesthetic management in surgical settings.

### 2.3. Eligibility Criteria

Studies were eligible if they evaluated magnesium sulphate administered in the perioperative setting within the defined population and context. Eligible evidence sources included randomised controlled trials, cohort studies, case-control studies, systematic reviews, meta-analyses, scoping reviews, and narrative reviews. Evidence syntheses and the narrative literature were included to capture mechanistic and conceptual evidence relevant to perioperative neuroprotection. No restrictions were applied regarding publication year or language.

Studies were excluded if they involved non-human experimental models, in vitro designs, or magnesium preparations other than magnesium sulphate when the specific pharmacological effect could not be isolated. Studies focusing exclusively on non-perioperative indications, such as treatment of eclampsia outside surgical care, acute asthma management, or nonsurgical arrhythmia treatment, were excluded. Editorials, commentaries, letters, and conference abstracts without full reports were also excluded. This exclusion was intentional and consistent with the translational orientation of the review: the objective was not to characterise preclinical mechanisms de novo but rather to evaluate how established pharmacological properties of magnesium sulphate are expressed and applied within the clinical perioperative context. Basic science evidence was thus treated as background rationale rather than as eligible evidence to be mapped.

### 2.4. Information Sources and Search Strategy

The literature search was conducted in four electronic databases: PubMed, Scopus, the Cochrane Library, and ProQuest, covering the period from inception to 15 January 2026. For the purposes of this review, the perioperative period was operationally defined as the continuum encompassing preoperative preparation, intraoperative anaesthetic management, and the immediate postoperative recovery phase up to and including hospital discharge. No language restrictions were applied during the search, although all included studies were published in or had full text available in English. The search strategy combined controlled vocabulary terms and free text keywords related to magnesium sulphate, general anaesthesia, perioperative care, neuroprotection, neuroinflammation, and endothelial dysfunction. Additional perioperative literature was captured through broader perioperative and intraoperative search terms. Boolean operators were applied to integrate magnesium-related terms with anaesthesia and mechanistic outcome domains. The full database-specific search strategies are provided in Supplementary Table S2.

### 2.5. Selection of Sources of Evidence

All retrieved records were imported into a reference management system, and duplicate records were removed prior to screening. Study selection was conducted in two sequential stages according to the predefined Population–Concept–Context eligibility criteria. In the first stage, titles and abstracts were screened to identify potentially relevant studies. In the second stage, full-text articles were assessed to determine final eligibility. The screening process was performed independently by two reviewers. Any discrepancies regarding study eligibility were resolved through discussion until consensus was achieved.

### 2.6. Data Charting

Data were extracted using a structured charting form developed for this review. Extracted variables included study design, population characteristics, perioperative setting, magnesium administration protocol, comparator groups, outcome domains evaluated, and key findings. For primary clinical studies, additional information regarding magnesium dosing regimens was recorded, including route of administration, loading dose, infusion rate, timing relative to anaesthetic induction, and duration of administration.

### 2.7. Evidence Classification and Synthesis

Each included study was classified according to the Oxford Centre for Evidence Based Medicine 2011 Levels of Evidence framework to contextualise the strength of evidence across heterogeneous study designs [24]. Given the methodological and clinical heterogeneity of the included literature, quantitative meta-analysis was not performed. Instead, findings were synthesised using evidence mapping approach. To improve interpretability across heterogeneous sources, the included literature was further categorised according to evidence type, distinguishing primary clinical evidence (randomised and observational clinical studies), synthesised evidence (systematic reviews and meta-analyses), and conceptual or mechanistic literature (narrative reviews and theoretical discussions). This classification allowed for differentiation between direct clinical findings, synthesised summaries of existing evidence, and conceptual mechanisms relevant to perioperative neuroprotection. It is acknowledged that the simultaneous inclusion of primary studies and evidence syntheses that potentially incorporate the same primary trials introduces a risk of evidential overlap. To mitigate this, evidence from synthesised sources (systematic reviews and meta-analyses) was used exclusively to characterise the breadth and consistency of existing findings across populations and settings, while primary clinical studies were interpreted as independent data sources contributing directly to the domain-based evidence mapping. Signals reported by both a primary study and a subsequent synthesis were noted as corroborating rather than additive evidence, and no study was counted more than once within any single outcome domain.

Studies were grouped according to predefined outcome domains and categorised according to their proximity to direct neuroprotective outcomes, including direct clinical evidence, indirect clinical evidence, biomarker-related evidence, and conceptual mechanistic evidence. These four domains were operationally defined as follows: Direct clinical evidence referred to studies evaluating validated neurological endpoints, including cognitive function, delirium, cerebral oxygenation, or neurovascular outcomes such as stroke or vasospasm. Indirect clinical evidence comprised studies measuring perioperative outcomes biologically relevant to neural stress but not constituting neurological endpoints per se, including postoperative pain, opioid consumption, emergence agitation, recovery quality, and shivering. Biomarker-related evidence included studies measuring biochemical or physiological surrogates associated with neuroinflammation or endothelial function, such as serum C-reactive protein levels. Conceptual mechanistic evidence encompassed narrative reviews and theoretical discussions addressing pharmacodynamic pathways relevant to neuroprotection, including NMDA receptor modulation and calcium channel regulation, without reporting primary clinical outcome data.

### 2.8. Analytical Environment

Data management, tabulation, and evidence mapping were performed using the R statistical computing environment (R Foundation for Statistical Computing, Vienna, Austria), using the current stable release R version 4.5.3 [25]. The analytical workflow included structured data processing, domain-based categorisation, and generation of descriptive evidence mapping outputs.

### 2.9. Risk-of-Bias Assessment

To address the methodological quality of the primary clinical studies, a formal risk-of-bias assessment was conducted. The Cochrane Risk of Bias 2 (RoB 2) tool was utilised for the randomised controlled trials, evaluating domains including random sequence generation, allocation concealment, blinding of participants, personnel, and outcome assessors, incomplete outcome data, and selective reporting. For the retrospective matched case-control study, methodological quality was appraised using the Newcastle–Ottawa Scale (NOS), focusing on selection, comparability, and exposure/outcome assessment. Conceptual and narrative reviews included in this scoping review were not formally appraised for risk of bias, as domain-specific tools are not applicable to these study designs.

## 3. Results

### 3.1. Study Selection

**Figure 1:**
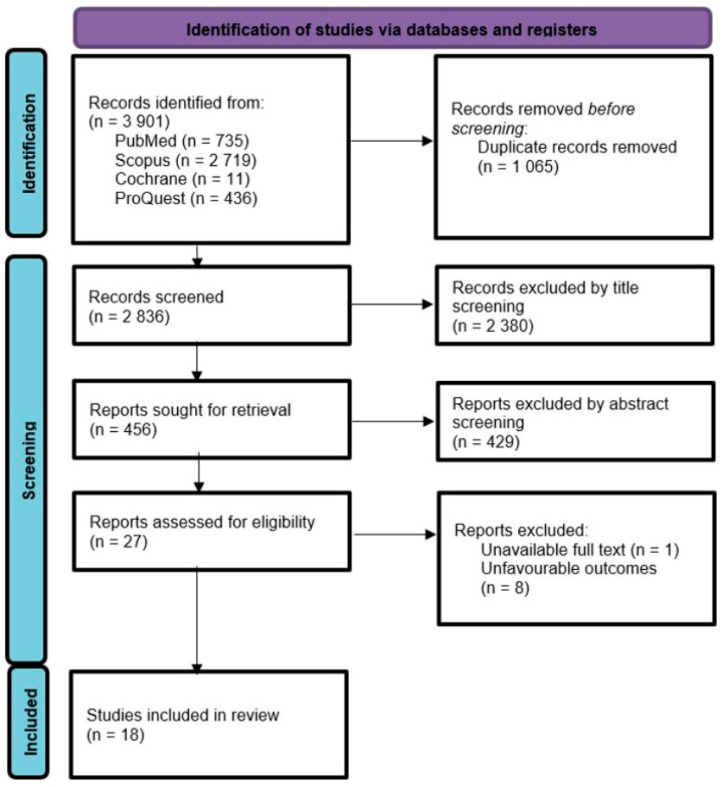
PRISMA flow diagram of the included studies.

The database search identified 3901 records from four databases, including Scopus (n = 2719), PubMed (n = 735), the Cochrane Library (n = 11), and ProQuest (n = 436). After the removal of duplicates and screening of titles and abstracts, potentially relevant studies underwent full-text assessment according to the predefined inclusion and exclusion criteria. Full-text exclusions were primarily due to non-perioperative contexts, absence of magnesium sulphate exposure, non-human experimental designs, or lack of relevant clinical or mechanistic outcomes. A total of 18 studies were ultimately included in the final synthesis. The study selection process is illustrated in Figure 1.

### 3.2. Characteristics of Included Studies

The characteristics of the included studies are summarised in Table 1. Among the 18 included studies, 4 were systematic reviews with meta-analyses, 8 were randomised controlled trials, 1 was a retrospective matched case-control study, 1 was a cross-sectional survey, and 4 were narrative reviews.

The randomised trials included sample sizes ranging from 60 to 327 participants and were conducted across diverse surgical settings including thoracic surgery, spinal surgery, breast cancer surgery, neurosurgical procedures, and mixed surgical populations [20,21,26,27,28,29,30,31]. One observational study involving 48 patients evaluated neurovascular outcomes in aneurysmal subarachnoid haemorrhage [32]. Systematic reviews and narrative reviews synthesised broader mechanistic and perioperative evidence regarding magnesium mediated physiological effects [1,11,34,35,36,37,38,39]. Across the included studies, magnesium sulphate was primarily evaluated as an adjuvant during perioperative anaesthetic management, most commonly administered via the intravenous route.

### 3.3. Magnesium Administration Protocols

Magnesium administration regimens are summarised in Table 2. Across nine primary clinical studies reporting quantitative dosing protocols, magnesium sulphate was consistently administered intravenously. Most perioperative trials used bolus plus infusion regimens, typically consisting of loading doses of 30 to 40 mg per kilogram followed by infusion rates of 10 to 15 mg per kilogram per hour during surgery [21,27,28,31]. Single bolus protocols of 50 mg per kilogram were used in two studies [20,29]. In contrast, neurocritical care studies employed prolonged infusion protocols lasting 10 to 14 days to maintain therapeutic magnesium levels following aneurysmal subarachnoid haemorrhage [26,32].

Mechanistic signals identified across studies are summarised in Table 3. These signals were interpreted according to their proximity to direct neuroprotective outcomes, distinguishing between direct neurological endpoints, indirect clinical proxies, biomarker-based evidence, and conceptual mechanistic hypotheses. Indirect clinical evidence supporting analgesic modulation was reported in six studies, with all demonstrating reductions in postoperative pain or opioid consumption [21,28,31,34,35,36].

Emergence or recovery outcomes were evaluated in four studies, with three reporting favourable effects such as reduced emergence agitation or improved recovery characteristics [20,21,31,35]. Postoperative shivering prevention showed consistent benefit in two studies [29,34]. In contrast, direct neurovascular outcomes demonstrated heterogeneous results. Among four studies evaluating neurological endpoints, only one study reported a favourable signal, indicating reduced vasospasm and delayed cerebral ischaemia [32], while others reported negative or inconclusive findings [26,39]. Biomarker-related evidence was limited, with only one trial evaluating inflammatory markers, which did not demonstrate consistent anti-inflammatory effects [30].

### 3.4. Integrated Evidence Landscape

**Figure 2:**
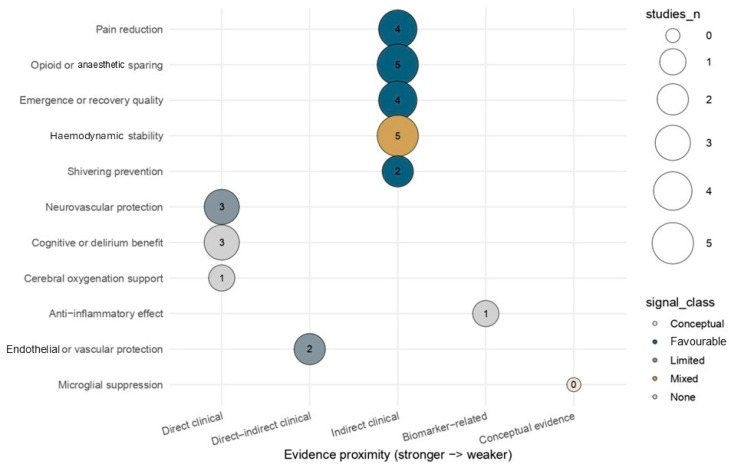
Evidence mapping of perioperative magnesium sulphate effects across neuroprotection-related domains.

The integrated evidence mapping across outcome domains is summarised in Table 4 and illustrated in Figure 2. Pain reduction demonstrated the most consistent signal, with 4 out of 4 studies reporting favourable outcomes [21,26,28,31]. Opioid or anaesthetic sparing effects were observed in 4 out of 5 studies [21,28,30,34,35]. Emergence or recovery quality demonstrated three favourable signals among four evaluating studies [20,21,31,35].

Haemodynamic stability showed inconsistent evidence, with one favourable signal among five studies [27,29,30,35,38]. Neurovascular protection showed limited evidence, with one supportive study among four evaluating studies [26,32,37,39]. Cognitive- or delirium-related outcomes demonstrated no consistent benefit across three studies [1,27,31].

Other mechanistic domains, including cerebral oxygenation support and anti-inflammatory signalling, were evaluated in single studies and did not demonstrate consistent effects [28,30]. Overall, the mapped evidence landscape demonstrates a consistent pattern of indirect perioperative benefits associated with magnesium administration, particularly in postoperative pain reduction, opioid sparing, and recovery characteristics. In contrast, direct neuroprotective outcomes such as neurological recovery, cerebral oxygenation, or neurovascular complications remain supported by limited and heterogeneous evidence.

### 3.5. Conceptual Mechanistic Synthesis

**Figure 3:**
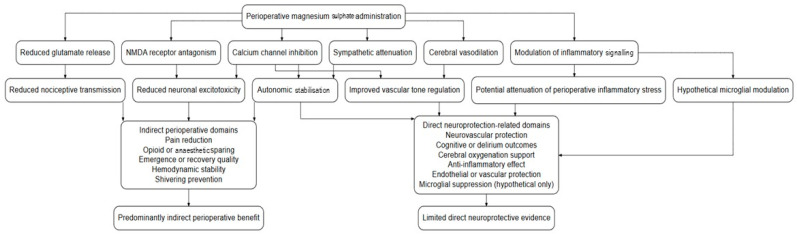
Conceptual mechanistic pathways linking perioperative magnesium sulphate administration to potential neuroprotective effects.

The proposed mechanistic integration of these findings is illustrated in Figure 3. The conceptual framework links perioperative magnesium administration with potential neuroprotective pathways including NMDA receptor antagonism, modulation of calcium mediated neuronal excitability, reduction of perioperative nociceptive burden, attenuation of sympathetic stress responses, and potential endothelial stabilisation. Although several pathways remain supported primarily by indirect clinical evidence or mechanistic plausibility, the overall pattern suggests that magnesium mediated neuroprotection may arise mainly through modulation of perioperative neural stress and nociceptive signalling. This interpretation aligns with the predominance of indirect clinical signals identified in the evidence mapping analysis. These mechanistic pathways represent a plausible biological framework rather than a confirmed neuroprotective mechanism, and their clinical significance awaits validation through dedicated endpoint-driven trials.

### 3.6. Methodological Quality and Risk of Bias

Formal methodological quality and risk-of-bias assessments were independently conducted for the nine primary clinical studies. Among the eight Randomised Controlled Trials (RCTs) evaluated using the Cochrane Risk of Bias 2 (RoB 2) tool, six studies ([20,21,26,27,29,30]) were rated as having a Low risk of bias. These studies generally demonstrated rigorous computer- or Internet-based random sequence generation, adequate allocation concealment using opaque sealed envelopes, successful double-blinding protocols for both personnel and outcome assessors, and an absence of significant selective reporting or missing outcome data. Two RCTs were classified as having Some Concerns [28] had a potential attrition bias due to the post-randomisation exclusion of 11 patients resulting from massive bleeding or prolonged surgery times, while [31] lacked sufficient methodological details regarding the exact processes used for random sequence generation and allocation concealment. The single retrospective matched case-control study [32] was evaluated using the NOS framework for observational studies and was deemed to have a Moderate risk of bias. Although this study robustly minimised confounding factors through an adequate case-control matching process, its retrospective design inherently carries a moderate risk of selection and information bias.

## 4. Discussion

This scoping review indicates that the putative neuroprotective value of perioperative magnesium sulphate is supported more consistently by indirect perioperative outcomes than by direct neurological endpoints [16,19]. The most recurrent favourable signals were observed in postoperative pain reduction, opioid or anaesthetic sparing, smoother emergence or early recovery, and shivering prevention, whereas evidence for cognitive protection, cerebral oxygenation support, anti-inflammatory effects, and neurovascular benefit remained limited or inconsistent [26,28,30]. Taken together, these findings suggest that magnesium may act less as a conventional organ specific neuroprotectant and more as a broader modulator of neural stress, nociceptive burden, and physiological instability during the perioperative period. This distinction is clinically important, attributing neuroprotective properties to magnesium sulphate on the basis of analgesic and recovery outcomes alone risks overstating the available evidence, and such claims should be interpreted within the acknowledged limitations of surrogate endpoint inference [1,10,11].

This interpretation is consistent with contemporary concepts of perioperative brain health, which frame postoperative brain injury as a distributed process involving neuroinflammation, synaptic dysfunction, endothelial activation, altered neurovascular coupling, and blood–brain barrier disruption rather than a single discrete insult [5,40]. Within this framework, an intervention may still provide clinically meaningful protection even when it does not consistently improve hard outcomes such as delirium, stroke, or long-term cognition [3,4,41]. This distinction matters because definitive neurocognitive outcomes generally require rigorous phenotyping, adequate sample size, and longer follow-up than most perioperative pharmacological trials have been designed to provide [3,6,41].

In that context, the repeated analgesic and opioid sparing effects identified in this review should not be regarded as merely supportive findings. Postoperative pain, excess opioid exposure, sleep disruption, agitation, and sympathetic overactivation are biologically relevant contributors to brain vulnerability because they can amplify inflammatory stress, impair recovery physiology, and promote delirium prone states [5,9,41]. The consistency with which magnesium reduced pain and opioid requirements across several surgical settings therefore strengthens the mechanistic rationale for further investigation, although these indirect signals should not be conflated with demonstrated neuroprotective efficacy in the absence of concurrent neurological endpoint assessment [10,18].

A similar argument applies to recovery-related outcomes. Recent synthesis suggests that intravenous magnesium may improve global postoperative recovery quality, while contemporary randomised trials in thoracoscopic lobectomy and radical mastectomy reported reduced emergence agitation and better early recovery-related outcomes [19,20,21]. These findings raise the possibility that magnesium attenuates neural stress during emergence, a vulnerable interval characterised by nociceptive surges, catecholamine activation, airway stimulation, and dysregulated wake transition [42,43]. Although recovery quality is not synonymous with neuroprotection, it may serve as a clinically relevant bridge between mechanistic plausibility and overt neurological outcomes [19].

The mechanistic basis for this interpretation, while biologically plausible, remains largely derived from pharmacodynamic evidence and indirect clinical signals rather than direct neuroprotective endpoint data. Magnesium modulates N-methyl-D-aspartate receptor activity and calcium influx, both of which are central to excitotoxicity, neuronal hyperexcitability, opioid induced hyperalgesia, and central sensitisation [11,12,34]. It also appears to influence sympathetic tone, vascular reactivity, and endothelial biology, while broader magnesium physiology has been linked to inflammatory regulation and nitric oxide-related pathways [38]. These properties are especially relevant to surgery because excitatory stress, inflammatory activation, vascular dysregulation, and autonomic disturbance usually unfold concurrently rather than in isolation [1,2,5].

This multimodal biology may explain why magnesium shows greater consistency in pain, opioid sparing, shivering prevention, and emergence modulation than in narrowly defined neurovascular or cognitive outcomes [16]. A drug that reduces nociceptive input, limits remifentanil-related hyperalgesia, blunts sympathetic stress responses, and supports vascular stability may lower the overall perioperative injury burden without producing a large isolated effect on one endpoint such as postoperative delirium [34,38]. This reading is also consistent with reviews describing magnesium as an adjuvant with both analgesic and haemodynamic relevance rather than as a single-purpose pharmacological adjunct [10,44].

At the same time, the limited direct neurological evidence should be interpreted cautiously rather than as simple proof for or against efficacy. The randomised trial in aneurysmal subarachnoid haemorrhage did not show improved neurological outcome with prolonged magnesium infusion, whereas a more recent matched case-control study suggested lower rates of vasospasm and delayed cerebral ischaemia with early administration [26,32]. Similarly, the meta-analysis in non-cardiac surgery found no consistent reduction in perioperative stroke or neurological complications, while a more recent neuroanaesthesia-focused synthesis described potentially favourable signals in selected neurocritical and neuroanaesthetic settings [1]. These discrepancies suggest that the more relevant question is not whether magnesium is universally neuroprotective but under which biological context, timing window, dosing strategy, and endpoint structure its effects become clinically detectable.

Methodological heterogeneity almost certainly contributes to the gap between indirect and direct outcomes. Most available trials were small single-centre studies with heterogeneous surgical populations, variable comparator regimens, and limited neurological phenotyping [19,28,36]. Cognitive outcomes were infrequently assessed using contemporary perioperative neurocognitive frameworks, biomarker sampling was sparse, and follow-up was usually short [41]. Magnesium regimens also varied markedly, ranging from single bolus strategies to bolus plus infusion protocols and prolonged neurocritical care infusions [26]. This degree of heterogeneity supports evidence mapping and cautions against treating the entire literature as though it addressed one unified efficacy question.

An additional implication of this review is that the field may be measuring the wrong outcomes or measuring the right outcomes at an inappropriate level [5]. If magnesium primarily reduces excitatory stress, opioid burden, sympathetic reactivity, and vascular instability, its earliest and most reliable clinical signals are more likely to emerge in domains such as pain, agitation, shivering, and recovery quality rather than in coarse endpoints such as stroke or dichotomised delirium [19,42]. This perspective does not weaken the neuroprotective hypothesis but instead reframes it within a multidimensional and temporally structured model of perioperative brain injury [11]. Future studies should therefore adopt hierarchical outcome frameworks integrating proximal biological markers, intermediate physiological phenotypes, and clinically meaningful neurocognitive endpoints to better capture treatment effects across the injury continuum.

The limited biomarker evidence in the present review warrants careful interpretation. Only one included study directly assessed inflammatory markers and did not demonstrate a consistent anti-inflammatory effect [30]. However, this absence of signal should not be interpreted as evidence of biological inactivity, as perioperative neuroinflammation is both temporally dynamic and compartmentalised, and may not be adequately captured through sparse peripheral sampling [8,45].

A similar limitation applies to cerebral oxygenation and endothelial domains. A clinical trial in traumatic brain injury demonstrated no improvement in regional cerebral oxygen saturation despite favourable analgesic effects [28]. Nonetheless, endothelial and microvascular endpoints remain largely underexplored in perioperative magnesium studies, despite increasing recognition of blood–brain barrier dysfunction and microvascular injury as central components of perioperative neurocognitive disorders [9,46]. This suggests that null findings in current studies may reflect limitations in measurement sensitivity rather than absence of biological effect.

From a clinical standpoint, these findings position magnesium sulphate as a multimodal adjunct rather than a definitive standalone neuroprotective agent. Its most consistent and clinically defensible role lies in improving perioperative recovery through analgesic and opioid sparing effects, with additional benefits in shivering prevention and emergence modulation [19,42]. While these effects are indirect, they remain clinically meaningful given the cumulative contribution of perioperative stress to neurological vulnerability [5]. However, current evidence remains insufficient to support magnesium as a targeted strategy for preventing postoperative delirium or long-term neurocognitive decline [3].

This review has several strengths. Its primary contribution lies in the translational framing and the explicit separation between direct neurological outcomes, indirect clinical proxies, and mechanistic evidence. This approach allows consistent signals such as pain reduction and opioid sparing to be interpreted appropriately without overstating their role as definitive neuroprotective endpoints.

Several limitations should also be acknowledged. The available evidence base remains relatively small and heterogeneous, with most studies conducted in single-centre settings and employing variable outcome definitions and dosing strategies. Neurological endpoints were inconsistently assessed and often underpowered, while mechanistic biomarker data were sparse. In addition, the predominance of intravenous administration limits inference regarding alternative delivery strategies or exposure-dependent effects. As with any scoping review, this study is designed to map evidence rather than provide pooled estimates and therefore cannot establish definitive efficacy.

Regarding methodological quality, we conducted a formal risk-of-bias assessment specifically for the primary clinical studies using the RoB 2 and NOS frameworks, which demonstrated that the majority of our primary evidence carries a low-to-moderate risk of bias. However, a formal critical appraisal using tools like AMSTAR-2 was not performed for the included systematic and narrative reviews, consistent with the scoping review methodology in which evidence mapping across heterogeneous literature types is the primary objective. Furthermore, the predominance of intravenous administration limits inference regarding alternative delivery strategies or exposure-dependent effects.

A further and particularly important limitation concerns the breadth of surgical and clinical contexts represented across the included studies. The included literature spans heterogeneous perioperative settings including neurocritical care following aneurysmal subarachnoid haemorrhage, intracranial tumour surgery, thoracoscopic lobectomy, radical mastectomy, lumbar fixation, spinal anaesthesia, and mixed surgical populations [20,21]. These settings differ substantially in terms of the nature and intensity of the surgical insult, the expected magnitude of perioperative neural stress, baseline patient vulnerability, and the biological plausibility of a pharmacological neuroprotective effect. Consequently, evidence signals identified in non-neurological surgical populations, such as analgesic benefits following thoracic or breast surgery, may not be directly translatable to neurologically vulnerable populations such as those undergoing cranial or neurovascular procedures. This heterogeneity of context is an inherent feature of the scoping review methodology and reflects the breadth of the mapping objective, but it necessarily limits the specificity and transferability of the translational conclusions drawn. Future work should stratify perioperative magnesium research according to clinical context, as the conditions under which magnesium may exert meaningful neuroprotective effects are likely to differ substantially across surgical specialties.

Future research should move beyond binary questions of efficacy and instead focus on identifying the clinical contexts and biological windows in which magnesium is most likely to exert meaningful effects. Large multicentre trials should prioritise high risk populations and incorporate multidomain outcome frameworks that capture both mechanistic and clinical endpoints across the perioperative trajectory.

## 5. Conclusions

The current evidence landscape supports magnesium sulphate as a biologically plausible and clinically promising perioperative adjunct whose strongest signals lie in modulation of pain, opioid exposure, recovery quality, and selected physiological stress responses rather than in definitive direct neurological protection. On the basis of the available evidence, perioperative magnesium sulphate should therefore be regarded as an agent that optimises the perioperative neural environment through indirect mechanisms and not as a pharmacologically validated neuroprotective agent in the classical sense. This distinction between perioperative optimisation and true neuroprotection is not merely semantic; it reflects a fundamental difference in the level of evidence required and the biological mechanisms that must be demonstrated before clinical recommendations for neuroprotective use can be justified.

The absence of consistent benefit on cognition, cerebral oxygenation, or major neurovascular outcomes should not be taken as proof of inefficacy but rather as an indication that the field has not yet aligned its mechanistic hypotheses, outcome selection, and trial design with the distributed biology of perioperative brain injury. On present evidence, magnesium is best understood as a candidate translational neuroprotective modulator with meaningful perioperative benefits and a clear rationale for more targeted, multidomain, and mechanism informed investigation.

## Figures and Tables

**Table 1:** Characteristics of included studies.

Study	Design	Population	Clinical Setting	Magnesium Exposure	Comparator	Outcome Domains Evaluated	Main Findings	Evidence Level
Primary Study								
Wong, 2010 [26]	Randomised controlled trial	Patients with aneurysmal subarachnoid haemorrhage	Neurocritical care	Intravenous infusion	Placebo	Neurovascular protection	No improvement in neurological outcomes	Level 2
Jitsinthunun, 2022 [27]	Randomised controlled trial	Adult patients	Meningioma craniotomy	Intravenous bolus plus infusion	Saline	Cognitive or delirium outcomes; haemodynamic stability	No improvement in cognitive outcomes or haemodynamic stability	Level 2
Sohn, 2022 [28]	Randomised controlled trial	Patients with mild traumatic brain injury	Surgery under general anaesthesia	Intravenous bolus plus infusion	Saline	Cerebral oxygenation support; pain reduction; opioid or anaesthetic sparing	No improvement in cerebral oxygenation; reduced postoperative pain and opioid consumption	Level 2
Pu, 2025 [20]	Randomised controlled trial	Adult surgical patients	Thoracoscopic surgery	Intravenous bolus	Placebo	Emergence or recovery quality; pain reduction	Reduced emergence agitation and postoperative pain	Level 2
Su, 2023 [21]	Randomised controlled trial	Breast cancer patients	Radical mastectomy	Intravenous bolus plus infusion	Placebo	Emergence or recovery quality; pain reduction; opioid or anaesthetic sparing	Reduced emergence agitation, postoperative pain, and remifentanil use	Level 2
Low, 2022 [29]	Randomised controlled trial	Adult surgical patients	Spinal anaesthesia	Intravenous bolus	Dose comparison	Shivering prevention; haemodynamic stability	Reduced postoperative shivering	Level 2
Etezadi, 2014 [30]	Randomised controlled trial	Adult neurosurgical patients	Intracranial tumour surgery	Intravenous infusion	Saline	Haemodynamic stability; opioid or anaesthetic sparing; anti-inflammatory effect	Reduced anaesthetic requirement and blood loss; no consistent anti-inflammatory effect	Level 2
Fathy, 2024 [31]	Randomised controlled trial	Adult surgical patients	Lumbar fixation surgery	Intravenous bolus plus infusion	Standard anaesthesia	Pain reduction; cognitive or delirium outcomes; emergence or recovery quality	Reduced postoperative pain and insomnia; no clear delirium reduction	Level 2
Feulner, 2024 [32]	Retrospective matched case-control study	Patients with aneurysmal subarachnoid haemorrhage	Neurovascular management	Intravenous infusion	No magnesium	Neurovascular protection	Reduced vasospasm and delayed cerebral ischemia	Level 3
Cavalcanti, 2019 [33]	Cross-sectional survey	Anaesthesiologists	Clinical practice	Mixed perioperative use	None	Pain reduction; opioid or anaesthetic sparing; safety reporting	Magnesium frequently used for analgesia and anaesthetic sparing in clinical practice	Level 4
Secondary Study								
Wu, 2025 [34]	Systematic review and meta-analysis	Adult surgical patients	General anaesthesia	Intravenous bolus plus infusion protocols	Placebo	Pain reduction; opioid or anaesthetic sparing; shivering prevention	Reduced postoperative pain, opioid consumption, shivering, and postoperative nausea and vomiting	Level 1
Jin, 2025 [35]	Systematic review and meta-analysis	Adult surgical patients	Spinal surgery	Intravenous bolus plus infusion protocols	Placebo or standard care	Pain reduction; opioid or anaesthetic sparing; emergence or recovery quality	Reduced postoperative pain and opioid consumption; improved recovery quality	Level 1
Campos, 2024 [36]	Systematic review and meta-analysis	Adult surgical patients	Spinal surgery	Intravenous bolus plus infusion protocols	Placebo or alternative analgesia	Pain reduction; opioid or anaesthetic sparing	Reduced postoperative pain and opioid consumption	Level 1
Zeng, 2018 [37]	Systematic review and meta-analysis	Adult surgical patients	Non-cardiac surgery	Mixed perioperative magnesium protocols	Placebo	Neurovascular protection	No consistent reduction in perioperative stroke or neurological complications	Level 1
Dahake, 2024 [11]	Narrative review	Surgical patients	Perioperative care	Mixed perioperative use	Various	Conceptual mechanisms of neuroprotection	NMDA receptor antagonism and calcium channel modulation discussed	Level 5
Sawant, 2024 [38]	Narrative review	Surgical patients	ENT surgery	Mixed perioperative use	Various	Haemodynamic stability	Possible attenuation of sympathetic response to intubation	Level 5
Chakane, 2021 [39]	Narrative review	Cardiac surgery patients	Cardiac surgery	Mixed perioperative use	Various	Neurovascular protection; cognitive or delirium outcomes	Evidence for neuroprotection remains inconclusive	Level 5
Bilotta, 2013 [1]	Narrative review	Surgical patients	Perioperative neuroprotection	Mixed perioperative use	Various	Cognitive or delirium outcomes	Possible reduction in postoperative neurological complications	Level 5

ENT: ear, nose, and throat.

**Table 2:** Detailed characteristics, administration protocols, and clinical outcomes of magnesium sulphate interventions in the included primary clinical studies.

Study and Design	Magnesium Protocol and Comparator	Primary Outcome	Secondary Outcome(s)	Scales Used and Measurement Time	Main Quantitative Result	Adverse Events (AEs)	Risk of Bias
Wong, 2010 [26] (RCT)	Mg: IV serum-targeted infusion (titrated to double baseline); within 48 h of diagnosis, duration 10–14 days. Control: Saline placebo.	Neurovascular protection/favourable neurological outcome.	Vasospasm incidence.	Scale: GOSE and mRS. Time: 6 months post-op.	No significant difference in favourable neurological outcome (GOSE 5–8: 64% in Mg vs. 63% in control group).	Infusion stopped in 3 pts (1%) due to severe limb weakness, hypocalcaemia, or hypernatraemia. Hypotension: 15% (Mg) vs. 13% (control) (*p* > 0.05).	Low
Etezadi, 2014 [30] (RCT)	Mg: IV 5 g infusion per session; given preoperative and intraoperative (total 3 sessions). Control: Saline.	Anti-inflammatory effect.	Haemodynamic parameters.	Scale: Serum CRP levels (mg/L). Time: Pre-op, 24 h, and 48 h post-op.	No significant difference in CRP between groups at 24 h (4.54 ± 1.45 vs. 5.67 ± 1.39, *p* = 0.243).	None specifically reported related to Mg toxicity; considered a safe anaesthetic adjuvant.	Low
Low, 2022 [29] (RCT)	Mg: IV 50 mg/kg bolus vs. 30 mg/kg bolus; administered after spinal block as a single dose over 20 min. Control: Active comparator (30 mg/kg Mg).	Prevention of shivering post-subarachnoid block.	Haemodynamic changes and rescue analgesic requirement.	Scale: 5-point shivering scale. Time: Highest grade from 15 min of infusion until discharge.	50 mg/kg group had significantly higher absence of shivering and required less rescue pethidine (10% vs. 32.5%, *p* = 0.026).	Incidence of hypotension was not significant (25% vs. 10%). No oxygen desaturation or bradycardia documented.	Low
Jitsinthunun, 2022 [27] (RCT)	Mg: IV 40 mg/kg bolus (30 min) + 10 mg/kg/h infusion; from skin incision until dural closure. Control: Saline.	Intraoperative blood loss.	Anaesthetic requirement and neurocognitive function.	Scale: Blood volume (mL), MoCA. Time: Intraoperative (blood loss); POD 3–7 (MoCA).	No significant difference in intraoperative blood loss (500 mL vs. 510 mL, *p* = 0.315) or MoCA scores.	No clinical signs of Mg toxicity. Post-op complications (seizure, CN palsy, delirium, and CSF leak) were similar between groups.	Low
Sohn, 2022 [28] (RCT)	Mg: IV 30 mg/kg bolus (10 min) + 15 mg/kg/h infusion; after intubation throughout surgery. Control: Saline.	Regional cerebral oxygen saturation (rSO2).	Post-op pain and analgesia.	Scale: NIRS (rSO2), NRS (pain). Time: Intraoperative (rSO2); 6–48 h post-op (NRS).	No significant difference in rSO2 decline. Pain intensity significantly lower in Mg group during first 6 h (6.8 vs. 8.2, *p* = 0.017).	One patient dropped out due to massive bleeding early in surgery. No unresolved critical desaturation events.	Some Concerns
Su, 2023 [21] (RCT)	Mg: IV 30 mg/kg bolus (first hour) + 10 mg/kg/h infusion; after induction until end of surgery. Control: Saline.	Incidence of emergence agitation (EA).	Postoperative pain.	Scale: Riker SAS, VAS. Time: Upon entering PACU (T0) until extubation.	Incidence of EA significantly lower in Mg group at T0 (OR 0.26, *p* = 0.011). Remifentanil consumption lower (300.4 vs. 559.3 µg, *p* < 0.001).	Safe profile; no significant major adverse events reported during emergence.	Low
Fathy, 2024 [31] (RCT)	Mg: IV 30 mg/kg bolus + 10 mg/kg/h infusion; given intraoperatively after induction. Control: Conventional anaesthesia.	Postoperative delirium and insomnia.	Predictors of outcomes.	Scale: MDAS, ISI, VAS. Time: MDAS (24–48 h post-op); ISI (pre-op and 2 weeks post-op).	Postoperative ISI, MDAS, and VAS scores were significantly lower in the Mg group (*p* < 0.05).	Careful monitoring showed no clinical signs of magnesium toxicity.	Some Concerns
Feulner, 2024 [32] (Retrospective Case-Control)	Mg: IV 50 mg/kg loading dose + continuous infusion (81 mmol/24 h); within 24 h of diagnosis for up to 14 days. Control: Nimodipine only.	Incidence of cerebral vasospasm (CV) and delayed cerebral ischaemia (DCI).	Functional outcome.	Scale: mRS. Time: 3 and 12 months post-treatment.	Incidence of CV and DCI was lower in patients receiving early IV MgSO_4_.	Risk profile is dose dependent; exact incidence of specific AEs not explicitly quantified but balanced with nimodipine.	Moderate
Pu, 2025 [20] (RCT)	Mg: IV 50 mg/kg bolus; administered 20 min before induction as a single dose. Control: Placebo.	Emergence agitation (EA).	Postoperative quality of recovery.	Scale: Riker SAS, QoR-40, and VAS. Time: During emergence (SAS); POD 1 + 2 (QoR-40).	EA incidence was significantly lower in Mg group (9.5% vs. 42.9%, *p* < 0.001). QoR-40 scores on POD 1 were significantly higher.	No specific adverse events or magnesium toxicity highlighted; reported as a safe adjunct.	Low

Notes: IV = Intravenous; AEs = Adverse Events; CRP = C-Reactive Protein; CSF = Cerebrospinal Fluid; CN = Cranial Nerve; CV = Cerebral Vasospasm; DCI = Delayed Cerebral Ischaemia; EA = Emergence Agitation; GOSE = Glasgow Outcome Scale Extended; ISI = Insomnia Severity Index; MDAS = Memorial Delirium Assessment Scale; MoCA = Montreal Cognitive Assessment; mRS = modified Rankin Scale; NIRS = Near-Infrared Spectroscopy; NRS = Numeric Rating Scale; PACU = Post-Anaesthesia Care Unit; POD = Postoperative Day; QoR-40 = Quality of Recovery-40; RCT = Randomised Controlled Trial; rSO2 = Regional Cerebral Oxygen Saturation; SAS = Sedation-Agitation Scale; VAS = Visual Analogue Scale. Mechanistic Evidence Signals.

**Table 3:** Neuroprotection-related mechanistic signals of MgSO_4_ across included studies.

Study	Evidence Proximity	Outcome Domain	Proxy Measure or Discussion	Signal Direction	Interpretation
Wu, 2025 [34]	Indirect clinical evidence	Pain reduction; opioid or anaesthetic sparing	Postoperative pain and opioid consumption	Supportive	Consistent perioperative analgesic modulation
Jin, 2025 [35]	Indirect clinical evidence	Pain reduction; emergence or recovery quality	Pain scores and recovery outcomes	Supportive	Analgesic benefit observed in spinal surgery
Campos, 2024 [36]	Indirect clinical evidence	Pain reduction	Postoperative pain and opioid consumption	Supportive	Supports perioperative antinociceptive effect
Zeng, 2018 [37]	Direct clinical evidence	Neurovascular protection	Stroke and neurological complications	Inconclusive	No clear neuroprotective benefit
Wong, 2010 [26]	Direct clinical evidence	Neurovascular protection	Vasospasm and functional outcome	Not supportive	No neurological improvement observed
Sohn, 2022 [28]	Direct-to-indirect clinical evidence	Cerebral oxygenation support	Regional cerebral oxygen saturation	Mixed	No oxygenation improvement despite analgesic benefit
Pu, 2025 [20]	Indirect clinical evidence	Emergence or recovery quality	Emergence agitation and recovery quality	Supportive	Suggests reduced perioperative neural stress
Su, 2023 [21]	Indirect clinical evidence	Emergence or recovery quality	Emergence agitation and opioid consumption	Supportive	Reduced nociceptive burden during emergence
Low, 2022 [29]	Indirect clinical evidence	Shivering prevention	Postoperative shivering	Supportive	Thermoregulatory modulation
Etezadi, 2014 [30]	Biomarker-related evidence	Anti-inflammatory effect	C-reactive protein levels	Mixed	No consistent anti-inflammatory signal
Fathy, 2024 [31]	Direct-to-indirect clinical evidence	Cognitive or delirium outcomes	Delirium and sleep disturbance	Mixed	Recovery improvement without clear neuroprotection
Feulner, 2024 [32]	Direct clinical evidence	Neurovascular protection	Vasospasm and delayed cerebral ischaemia	Supportive	Possible neurovascular benefit
Dahake, 2024 [11]	Conceptual evidence	Opioid or anaesthetic sparing	NMDA receptor antagonism	Supportive	Mechanistic plausibility for neuroprotection
Sawant, 2024 [38]	Conceptual evidence	Haemodynamic stability	Sympathetic response modulation	Supportive	Possible autonomic stabilisation
Chakane, 2021 [39]	Conceptual evidence	Neurovascular protection	Narrative synthesis	Inconclusive	Clinical evidence remains limited
Bilotta, 2013 [1]	Conceptual evidence	Cognitive or delirium outcomes	Narrative synthesis	Mixed	Possible neurological protection in selected contexts

NMDA, N-methyl-D-aspartate.

**Table 4:** Integrated clinical and mechanistic evidence landscape of MgSO_4_ in perioperative neuroprotection.

Outcome Domain	Evidence Proximity	Studies Evaluating Domain (n)	Evaluating Studies	Studies Reporting Favourable Signal (n)	Overall Mapped Signal	Main Interpretation
Pain reduction	Indirect clinical evidence	4	[20,21,28,31]	4	Recurrent favourable signal	Consistent perioperative analgesic benefit
Opioid or anaesthetic sparing	Indirect clinical evidence	5	[21,28,30,34,35]	4	Frequent favourable signal	Supports anaesthetic-sparing effect
Emergence or recovery quality	Indirect clinical evidence	4	[20,21,31,35]	3	Moderately consistent favourable signal	Suggests smoother postoperative recovery
Haemodynamic stability	Indirect clinical evidence	5	[27,29,30,35,38]	1	Inconsistent	Possible autonomic modulation but inconsistent evidence
Shivering prevention	Indirect clinical evidence	2	[29,34]	2	Recurrent favourable signal	Consistent anti-shivering effect
Neurovascular protection	Direct clinical evidence	4	[26,32,37,39]	1	Limited and conflicting direct evidence	Evidence heterogeneous across neurovascular contexts
Cognitive or delirium outcomes	Direct clinical evidence	3	[1,27,31]	0	No clear direct benefit	No consistent cognitive protection demonstrated
Cerebral oxygenation support	Direct clinical evidence	1	[28]	0	Not demonstrated	No improvement in cerebral oxygenation detected
Anti-inflammatory effect	Biomarker-related evidence	1	[30]	0	Not demonstrated	Human biomarker evidence remains limited
Endothelial or vascular protection	Direct-to-indirect clinical evidence	2	[30,32]	1	Limited possible signal	Possible vascular modulation
Microglial suppression	Conceptual evidence	0	—	0	Hypothetical only	Mechanistic hypothesis without clinical testing

MgSO_4_: magnesium sulphate.

## Data Availability

The original contributions presented in this study are included in the article/Supplementary Material. Further inquiries can be directed to the corresponding author.

## Supplementary Materials

The following supporting information can be downloaded at: https://www.mdpi.com/article/10.3390/jcm15135032/s1, Table S1: PRISMA-ScR checklist; Table S2: Search strategy.
